# Anatomical Structures Responsible for CTEV Relapse after Ponseti Treatment

**DOI:** 10.3390/children9050581

**Published:** 2022-04-19

**Authors:** Nikolaos Laliotis, Chrysanthos Chrysanthou, Panagiotis Konstandinidis, Nikolaos Anastasopoulos

**Affiliations:** 1 Orthopaedic Department, Inter Balkan Medical Center, Asklipiou 10 Pilea, 57001 Thessaloniki, Greece; chr.chrysanthou@gmail.com (C.C.); panos192003@gmail.com (P.K.); 2 Medical Department, Aristotelion University of Thessaloniki, 54124 Thessaloniki, Greece; sv2dsy@auth.gr

**Keywords:** club foot, Ponseti method, relapse club foot, paratenon thickening, anatomical structures

## Abstract

Relapse of deformity after a successful Ponseti treatment remains a problem for the management of clubfoot. An untreated varus heel position and restricted dorsal flexion of the ankle are the main features of recurrences. We analyze the anatomical structures responsible for these recurrences. Materials and methods: During 5 years, 52 children with CTEV (Congenital Talipes Equino Varus) were treated with casts according to the Ponseti method, with a mean number of 7 casts. Closed percutaneous tenotomy was performed in 28 infants. Children were followed monthly and treated with the continuous use of a molded cast. We had 9 children with relapsed clubfeet. During the standing and walking phase, they had a fixed deformity with a varus position of the heel and dorsal flexion of the ankle <10 d. They were surgically treated with the posterolateral approach. Results: In all patients, we found a severe thickening of the paratenon of the Achilles in the medial side, with adhesions with the subcutaneous tissue. The achilles after the previous tenotomy was completely regenerated. The achilles was medially displaced. Conclusions: A severe thickening of the paratenon of the achilles and adhesions with the subcutaneous tissue are anatomical structures in fixed relapsed cases of clubfoot. We treated our patients with an appropriate surgical release.

## 1. Introduction

The management of CTEV has completed changed since the introduction of the Ponseti method of treatment. Clubfoot remains with an unknown etiology, possibly due to genetic and environmental factors. The main concern is a relapse of the deformity, which is estimated to exceed even 50%. Ponseti stated that CTEV has an endogenous tendency to relapse, expressing the unknown pathogenesis of the deformity. The reason why corrected feet relapse after the Ponseti method is unknown. The severity of the deformity, the number of casts required for correction, the accuracy, and the compliance with appropriate orthotic treatment are factors related to the recurrence of the deformity [1,2,3,4,5,6].

In relapsed feet, the most important elements of the remaining deformity are the varus position of the hindfoot with the reduced dorsal flexion of the ankle.

In the previous decades when extensive surgical releases were performed, detailed descriptions of the various constricted anatomical structures were well described. Ippolito and Ponseti reported that there was fibrous tissue that penetrated the muscle, ligaments and tendon sheath in the posterior and medial side of the foot. Sano et al. in histological studies found myofibroblastic characteristics in specimens that contributed to the recurrence of the deformity. Turco found a dense mass of scar tissue that involved the medial structures [7,8,9,10,11].

Our study aims to analyze our surgical findings in children that had a relapse of CTEV after the initial Ponseti method. We present the thickening of the paratenon, which shifts the Achilles tendon in the medial position, as an anatomical factor responsible for the recurrence of the deformity. 

## 2. Materials and Methods

During the period 2014–2019, we treated 52 babies with CTEV, who are available for appropriate evaluation and whom we continuously followed up. This is a retrospective study, conducted from the same group of pediatric surgeons that started the initial treatment. We have included in this study only neonates that had an initial evaluation and started treatment in our department, excluding those that were referred after trials for treatment elsewhere. We excluded infants with CTEV related to syndromes, such as arthrogryposis or multiple deformities.

There were 37 males and 15 females, and 12 of them had a bilateral deformity. Each child had digital photo documentation on the three levels (frontal, lateral, plantar), and the varus ankle, the equinus of the ankle, and the varus foot were measured. Skin creases above the calcaneum and on the medial side of the plantar surface were recorded. Clubfeet were categorized into 3 groups, mild, moderate, and severe CTEV.

Casts were applied with the principles of the Ponseti method. We continued until we achieved the correction of the varus of the hindfoot, the correction of the forefoot and elimination of the skin creases. The number of casts required for correction varied from 5 to 11 (mean 7).

Those babies that remained with a restricted dorsal flexion of the ankle of less than 10° were examined with a lateral x-ray of the feet, in maximum dorsal flexion with a corrected hindfoot. The angle was measured as less than 20°. Thereafter, we performed a percutaneous tenotomy of the Achilles. This was done in a theater under sedation and with the application of a cast above the knee for 3 weeks. All procedures were performed from the same pediatric orthopaedic surgeon. Tenotomy was performed in 28 infants. For those with bilateral CTEV, tenotomy was done simultaneously for both feet. During the tenotomy, we could not achieve the appropriate correction of the equinus after the procedure in only 2 babies. They were classified as uncorrected clubfeet after the initial Ponseti treatment.

After removal of the POP, an individually molted cast below the knee was applied on the affected leg. Parents were performing stretching exercises of the foot at least 3 times a day. Patients were followed every month, with a particular observation of the use of the cast and the strict exercise program. 

Our patients were evaluated at the age of 8–10 months when they started to stand as well as in their first attempts to perform first steps by the age of one year. Evaluation of the foot was performed with a clinical examination and with digital photos. We measure the dorsal flexion of the ankle when the heel was in the neutral position. Infants with dorsal flexion >15 d with a neutral or valgus heel were considered as having a very good result.

In 10 infants and children (aged 9–18 months), the result was not satisfactory, with elements of reduced dorsal flexion <10 d, a varus position of the heel and a residual forefoot adduction. They were standing or walking with a varus heel and on the lateral border of the foot. In order to increase the dorsal flexion, the infant had to invert the foot. Xray on the lateral view was undertaken to measure the angle between the talus and calcaneum.

In two of them who were borderline upon examination with a flexible foot, we performed a second tenotomy of the Achilles. One of the two achieved a good result, while the second continued to have relapsed results, leaving a group of 9 children. In 2 children, recurrence was bilateral. After a thorough consultation with the parents, we proposed an open surgical procedure (Table 1.)

## 3. Results

**Figure 1:**
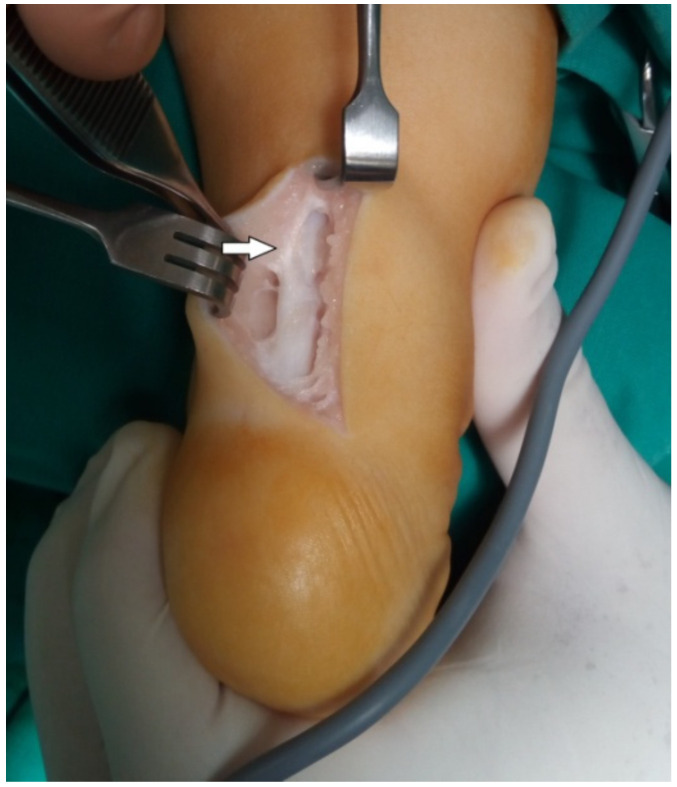
Arrow points to the adhesions of the thickened paratenon to the subcutaneous tissue.

**Figure 2:**
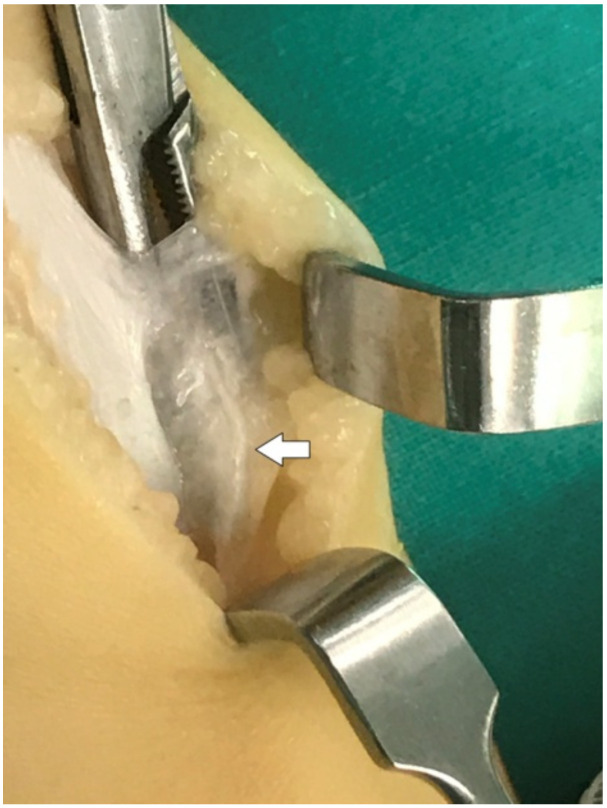
Arrow points to the adhesions of the thickened paratenon to the subcutaneous tissue.

Under GA (general anesthesia), in the prone position, with a tourniquet, we perform a posterolateral incision of 4 cm, ending at the middle space between the calcaneum and the lateral malleolus. After the skin and subcutaneous tissue incision, the Achilles sheath was identified. The small area of scar formation from the previously closed tenotomy was found. A striking observation was the severe thickening of the medial sheath of the Achilles, with adhesions to the subcutaneous fat. Incising the sheath, the tendon showed complete regeneration in the area of the previous tenotomy. We could not differentiate the structure of the incised area with the remaining tendon. The tendon sheath was markedly thickened in the medial side. This thickening was less remarkable in the lateral area of the Achilles. The insertion of the Achilles was found in the medial part of the calcaneum. The Achilles was shifted in the medial part of the calf, creating the varus position of the heel. This thickening mainly affected the paratenon sheath, leaving the neurovascular band within normal tissue. After opening the thickened medial sheath, the neurovascular band with the FDP was identified (Figure 1 and Figure 2).

We perform a Z type lengthening of the Achilles of 2.5–3 cm.

We proceed with the identification of the subtalar and ankle joint. We incised the thickened capsule starting from the lateral side, up to the lateral malleolus. The thickening of the peroneus sheath was less than that in the medial side of the Achilles tendon. We identified the sheath of the FHL, and we opened the sheath and performed an elongation of FHL in the musculotendinous junction. We completed the release with the opening of the capsule of the joints on the medial side. We reduced the talus by correcting the residual inclination and restoring the anatomy of the ankle joint. We used 1 K wire to stabilize the joint passing from the heel up to the tibial metaphysis. This was adequate to correct the elements of CTEV. In three children, we performed a lengthening of the tibialis posterior, because there was a remaining varus position of the foot. We completed the procedure with suturing of the elongated Achilles. We used 2–3 stitches between the lateral part of the paratenon of the Achilles, with the peroneal sheath, to improve the valgus position of the Achilles insertion.

We applied a POP above the knee for four weeks. We removed the K wire in 10 days and reapplied POP for the next 20 days. 

We regularly followed our patients. They were wearing an appropriate personalized molded cast for the foot when sleeping. All our patients were in the standing and walking period, so we trained their parents to supervise the standing and walking position of the feet and to correct any residual element of inversion of the foot. The program of stretching of the medial elements of the foot was continued. They were using a normal shoe, a type of boot. Physiotherapy was provided, whenever available, facilitating the correct position of the foot and further improving the appropriate walking of the children. None of these patients required any further surgical intervention, with a follow-up of 1–5 years.

## 4. Discussion

Since the introduction of the Ponseti method of treatment, there has been a remarkable improvement in our results, correcting all the elements of the clubfoot and ending with a flexible plantigrade foot [4,5,12].

The definition of corrected and relapsed clubfoot is not clearly defined. After the correction phase, feet must have a corrected varus heel that has achieved at least a neutral position. It is important for the foot to achieve a dorsal flexion of 15°, measured with a normal heel position, to achieve a good result. During the period when infants start the standing phase and start walking, we can evaluate the initial result of our treatment. Our relapsing incidence was 17%.

Bhaskar and Patni [13] proposed a classification for relapsed clubfoot. This is based on supination, adduction and reduced dorsiflexion of the foot, with elements of dynamic or fixed deformities. The most appropriate definition for recurrent deformity is the requirement for further treatment [3,4,13].

Several authors report a relapse of clubfoot deformation of up to 50% for patients treated with the appropriate Ponseti method. However, more than 50% of them will require another procedure later on, most commonly a tibialis anterior transfer [5].

Compliance with the strict post-tenotomy regime is considered the most important factor. However, it cannot explain all relapses. The bracing period is up to four years, even though further recurrence has been reported the more we have followed up with our patients. Bracing difficulties have been mentioned. The older the child, the more difficult it is to adhere to bilateral splints with the brace. There is no definition for non-compliance, but at least 10 hours of wearing the splint are required [13,14,15,16,17].

Zionts et al. report that all patients achieved an initial correction of the clubfoot deformity, but that despite the emphasis for a prolonged use of braces there was a relapse of the deformity of up to 68%, and 38% received an anterior tibial tendon transfer [6].

It is debatable whether the initial severity of the deformity produces relapses. The number of casts that are required for the correction of the deformity is implicated in the rate of recurrence and may be linked to the severity of the deformity [18,19,20,21]. There are different types of contractures and stiffness in the process of clubfoot management [5]. In our patients, we follow up with a strict program with a molded cast that they wear while sleeping, but without the bar of the Dennis Brown device, which we think is difficult to comply with. We follow a continuous program of exercises, appropriate casting and training to correct the position of the foot in the standing and walking phases.

The role of stretching of the medial structures that are stiff is well described. Sheta present an exercise protocol that eliminates the use of bracing, and they report excellent results with the Ponseti treatment [22]. The use of unilateral AFO has already been described, as well as more sophisticated braces to correct internal rotation [23,24].

There are several reports of very satisfying results after repeating casting and new tenotomy during relapses. Praag et al. report, with an adequate follow up, excellent results after recasting for feet that had a non-fixed foot that had a passive motion, indicating a response to passive motion. They report difficulties with boots and bars in older children and the use of articulating AFO during the daytime [25,26,27].

In our children for whom the initial results from the Ponseti method were not satisfied, we noticed the varus position of the heel and the tightness of the Achilles tendon, which could be palpated in the medial side of the ankle joint. It has been reported in residual or relapsed deformities that there is medialization of the insertion of the Achilles [14].

The lengthening of the Achilles alone is not sufficient to correct the clubfoot deformity. The concept of the Ponseti regime is to correct the varus position of the heel by initially inverting the 1st ray and then correcting the varus forefoot, before proceeding as a final step to correct the equinus, with a closed tenotomy. In the previous decades involving extensive surgical releases, there was no method of treatment that involved Achilles lengthening. 

There are many descriptions of the anatomic alterations of the complex deformity of clubfoot in the literature. In the period of extensive surgical releases involving the correction of the posterior part of the foot as well as of the medial side, almost all elements were reported to be thickened, and an extensive release of the joints and tendon elongations were performed to achieve a plantigrade foot. Unfortunately, severe stiffness often complicated the result, resulting in stiff and deformed feet. 

The Achilles has a false sheath that is referred to as the paratenon. The deep fascia of the leg is in contact with this sheath and fuses with it near the calcaneum, acting as a retinaculum for the tendon [28].

The foot of the newborn child is different to the adult one. The Achilles in the newborn is directly attached to the bone in a more varus position [29]. This was an important finding in our children with a relapsed clubfoot, and it was exacerbated by the thickening and adhesions of the paratenon, bringing the Achilles into a varus position.

Ponseti described that the deep and superficial fascia of the calf were thicker and that bundles of connective tissue fibers from the deep fascia penetrated into the muscle. The term retracting fibrosis was used for them, related to the etiology and the recurrence of CTEV. The severe thickness and shortening of the posterior tibial tendon and sheath and of the tibionavicular and calcaneonavicular ligament are well described in the pathological anatomy of severe clubfeet. In idiopathic clubfeet of aborted fetuses, the tibialis posterior, FHL and FDL were wrapped in a fibrotic mass. The Achilles tendon enters the clubfoot on the posteromedial supinated calcaneum. Ippolito described the wide insertion of the Achilles onto the posterior calcaneal tuberosity, receiving fibrous tissue strands from the ankle joint [8,11,30].

During the operation, we found that the thickened medial sheath of the Achilles adhered to the subcutaneous fat and shifted the tendon on the medial side, exacerbating the varus position of the heel. The tendon itself was found to be completely regenerated in the area of the previously performed tenotomy. This thickening extended from the calcaneum up to 4–5 cm proximally. This thickening mainly affected the paratenon, leaving the neurovascular band with its sheath with less thickening. This fibrosis of the sheath was less remarkable on the lateral side of the tendon and the peroneal sheath.

In MRI investigations of children with treatment-resistant clubfoot, Moon et al. described the presence of epimysial fat deposition and intramuscular fat deposition, different to atrophy of the muscles. This may correspond to the fibrosis of the paratenon and the severe adhesions to the subcutaneous tissue of our surgical findings [31]. Dietz et al. [32] stated that tissues in the posteromedial foot had less suppleness and that the deformity tended to recur even after an adequate initial correction. Treatment with joint invasive surgery increases with the duration of the follow-up [3].

We have treated children with relapsed clubfoot with a surgical procedure and not by repeating the Ponseti method with casts and a new tenotomy. A new attempt with casts and tenotomy was only made for two infants with a flexible deformity, and one of them succeeded in a nice correction. Our surgical procedure, with the release of the paratenon, lengthening of the Achilles, release of the ankle and subtalar joint, and lengthening of FHL, can achieve a good result with a straight plantigrade foot. In our patients aged 9–18 months, we found it essential for the complete correction of the deformity. 

A limitation of the study is that the evaluation of the results was performed by the same surgeon who used the Ponseti method and performed the tenotomy. The same person described the surgical finding of the thickening of the paratenon of the Achilles, which adhered to the subcutaneous tissue, along with the medial displacement of the Achilles. We have consistently used an individually moulded cast to maintain the correction, instead of the abduction orthosis. We have used a larger number of casts in the initial correction, achieving a better correction of the equinus deformity. This can explain the lower number of tenotomies that were performed in the first correction.

## 5. Conclusions

The Ponseti method of treatment is the standard approach for the initial management of CTEV. Our relapses are at 17%, with a remaining varus heel position and restricted dorsal flexion of the foot. We surgically treated these patients, who had a medialization of the Achilles with an extensive thickening of the paratenon that adhered to the subcutaneous tissue. We propose that this anatomical element is an important factor in relapsed clubfeet that require surgical treatment to improve the varus heel and the dorsal flexion of the ankle. 

## Figures and Tables

**Table 1:** Patient Characteristics.

Patient	Sex	Age at Operation	Side	Grade of Initial Deformation	Number of Casts
1	M	15 m	R	Moderate	8
2	M	14 m	R	Severe	5
3	M	9 m	Bilateral	Severe	11
4	F	18 m *	Bilateral	Moderate	9
5	F	10 m	L	Severe	7
6	M	14 m	L	Moderate	10
7	M	10 m	R	Severe	8
8	M	9 m	R	Moderate	8
9	M	15 m	L	Severe	7

* Patient with 2nd closed tenotomy.
